# Cutoff values for appendicular skeletal muscle mass and strength in relation to fear of falling among Brazilian older adults: cross-sectional study

**DOI:** 10.1590/1516-3180.2017.0049030517

**Published:** 2017-11-06

**Authors:** Ricardo Aurélio Carvalho Sampaio, Priscila Yukari Sewo Sampaio, Luz Albany Arcila Castaño, João Francisco Barbieri, Hélio José Coelho, Hidenori Arai, Marco Carlos Uchida, Gustavo Luis Gutierrez

**Affiliations:** I PhD. Physical Educator, Applied Kinesiology Laboratory, School of Physical Education, Universidade Estadual de Campinas (UNICAMP), Campinas (SP), Brazil.; II PhD. Occupational Therapist and Assistant Professor, Department of Occupational Therapy, Universidade Federal de Sergipe (UFS), Lagarto (SE), Brazil.; III Physical Educator, Department of Sports Science and Recreation, Universidad Tecnologica de Pereira, Pereira, Risaralda, Colombia.; IV Physical Educator and Master’s Stu­dent, School of Physical Education, Universidade Estadual de Campinas (UNICAMP), Campinas (SP), Brazil.; V MSc. Physical Educator and Doctoral Student, Applied Kinesiology Laboratory, School of Physical Education, Universidade Estadual de Campinas (UNICAMP), Campinas (SP), Brazil.; VI MD, PhD. Deputy Director, Kokuritsu Choju Iryo Kenkyu Center Kenkyujo (NCGG), Obu, Aichi Prefecture, Japan.; VII PhD. Associate Professor and Head of Applied Kinesiology Laboratory, School of Physical Education, Universidade Estadual de Campinas (UNICAMP), Campinas (SP), Brazil.; VIII PhD. Professor, School of Physical Education, Universidade Estadual de Campinas (UNICAMP), Campinas (SP), Brazil.

**Keywords:** Aging, Sarcopenia, Muscle, skeletal, Hand strength, Walking speed

## Abstract

**CONTEXT AND OBJECTIVE:**

Sarcopenia is an emerging public health issue in Brazil. Because of its high prevalence and the lack of national data, the objective here was to identify cutoff points for appendicular skeletal muscle (ASM) and handgrip strength in relation to fear of falling among Brazilian older adults.

**DESIGN AND SETTING:**

Cross-sectional study; in the community.

**METHODS:**

Participants underwent morphological and functional evaluations; and were asked about previous falls and fear of falling. Different adjustments to ASM and handgrip strength were used. Slow walking speed was defined as < 0.8 m/s or 1.0 m/s. Gender and age groups were compared using t tests, analysis of variance (ANOVA), chi-square test or Fisher’s exact test. Receiver operating characteristic curves were used to identify cutoffs for ASM and handgrip strength in relation to fear of falling.

**RESULTS:**

578 older adults participated in this study. Function levels decreased with increasing age, and body composition differed between the sexes. In relation to fear of falling, the cutoffs for ASM adjusted for body mass index (BMI) were < 0.85 for men and < 0.53 for women; the cutoffs for absolute handgrip strength and relative handgrip strength (adjusted for BMI) were 30.0 kgf and 21.7 kgf, and 1.07 and 0.66, for men and women, respectively.

**CONCLUSION:**

The values presented can be used as references in clinical practice and research. We recommend use of ASM adjusted for BMI and choosing between absolute and relative handgrip strength for men and women, according to study needs.

## INTRODUCTION

Sarcopenia, defined as progressive loss of muscle mass and strength/functionality with aging, is an emerging public health issue in Brazil.^1^ Loss of muscle mass and function may result in loss of physical capabilities (e.g. endurance, strength and muscle power), poor quality of life, unfavorable metabolic effects, falls and fear of falling, frailty and mortality among older adults. Sarcopenia is frequently associated with multiple morbid conditions, smoking habit, low body mass index (BMI), malnutrition and physical inactivity.^2^

Several consensuses and recommendations have been proposed by different institutions in attempts to standardize the conceptual approaches used to diagnose sarcopenia.^2^^,^^3^^,^^4^^,^^5^ Among these, experts agree that three key factors should be addressed: body composition (muscle mass), functionality (e.g. walking speed) and muscle strength (e.g. handgrip strength).

It has been estimated that after the age of 50, muscle mass decreases consistently at a rate of approximately 1% per year, walking speed at a rate of 2.0-2.2% and handgrip strength at a rate of 1.9-5.0%, as a result of the transition process of decreasing lean body mass and increasing fat accumulation.^6^^,^^7^ Cutoffs and reference values have also been presented in consensuses and recommendations. In addition to the international characteristics of the studies from which these values were compiled, most of these studies were conducted in developed countries and/or in countries that differ genetically, ethnically and culturally from Brazil. Moreover, even if the miscegenation of the Brazilian population has been taken into consideration, there are difficulties and limitations in making inferences from these values in relation to Brazilian older adults.

Great importance is now placed on sarcopenia, which recently culminated in determination of an International Classification of Diseases (ICD-10) code.^8^ Furthermore, the rising prevalence of sarcopenia among older Brazilians has now reached 17%,^1^ yet there is a lack of national preliminary data. Therefore, in this light, the aim of this study was to identify cutoff values for appendicular skeletal muscle (ASM), and handgrip strength in relation to fear of falling among Brazilian older adults.

## OBJECTIVE

The aim of this study was primarily to identify evidence-based cutoff values for ASM and handgrip strength in relation to fear of falling; and secondarily, to ascertain the morphological and functional characteristics of Brazilian older adults according to gender and age groups.

## METHODS

### Design and ethics

This study had a cross-sectional design (frequency study) and data were collected during 2015 and 2016.

The present study was approved by the Ethics Committee of the University of Campinas, under protocol #39437514.0.0000.5404. All participants signed an informed consent statement in which they agreed to participate in the study, and this was signed before data collection.

### Subjects

Subjects were recruited from four community centers for older adults in southeastern and southern Brazil during the study period, and were invited to participate voluntarily in a convenience sample. In these centers, they undertook social and physical activities provided by physical educators. Although subjects were recruited mainly in community centers, individuals who were not participating in regular activities (e.g, neighbors, relatives and others living in the community) were also invited to participate. This ensured variability of this sample. It is important to mention that neither center was responsible for rehabilitation or for any kind of medical treatment.

The inclusion criteria were that the subjects should be: a) community-dwelling individuals; b) 60 years of age or older; and c) able to answer questions and perform functional and body composition tests. The exclusion criteria comprised situations of: a) uncontrolled cardiovascular or pulmonary disease, conditions associated with a risk of falling (i.e. Parkinson’s disease or stroke), physical and cognitive impairment (according to reported chronic diseases, e.g. presence of conditions that might entail a requirement for assistance in basic activities of daily living) and items present in the functional assessment staging of Alzheimer’s disease (verified onsite); b) use of a metal prosthesis and/or pacemaker (i.e. interference with the bioelectrical impedance analysis).

### Assessments

The assessments were divided into two steps: a) indirect assessments based on questionnaires; and b) direct assessments based on morphofunctional evaluations (i.e. anthropometric characteristics and physical function). Before the evaluations, all tests were explained in detail to all participants by an experienced researcher. Verbal encouragement was provided to ensure that the participants reached the best performance possible.

### Indirect assessments

#### Chronic degenerative diseases, age, fear of falling and falls

A questionnaire was used to obtain data regarding the presence of chronic diseases, age, fear of falling and occurrences of falls during the year prior to this investigation. The questionnaire was based on simple questions that were answered by means of binary constructs (i.e. yes or no), so as to avoid possible misunderstanding between the researchers and the subjects. First, an extensive list of the most prevalent chronic diseases (e.g. hypertension, diabetes or osteoporosis) among older adults was presented to the participants. They then stated whether they had any previous clinical diagnosis of the chronic condition (in the form of a yes/no response). These data are not shown and were used solely for exclusion purposes.

In relation to fear of falling, the following question was asked: “Are you afraid of falling?”. The following question was asked about occurrences of falls: “Have you experienced a fall in the past year?’’. It is important to mention that only the question about occurrences of falls was retrospective: all the other questions and evaluations were in relation to the period within which the study was conducted.

### Direct assessments

#### Anthropometric measurements

Height was measured using a standard stadiometer and waist and hip circumferences using a measuring tape. The body composition was assessed by means of bioelectrical impedance analysis (BIA) (Tanita BC-108, Tokyo, Japan). The equipment provided the weight of the subject, and the height was inserted manually by the researcher. The analysis gave values for absolute and segmented muscle and fat mass. The Tanita BIA uses a frequency of 50 kHz to measure the quantity of intra and extracellular water in the body. This equipment has eight electrodes: four under the subjects’ feet and four on their hands. The ASM values (sum of muscle mass of limbs) are useful for diagnosing sarcopenia.^9^ In this study, we used several adjustments (i.e. according to BMI, height squared and weight), as well as the non-adjusted data, to ascertain the best approach for Brazilian older adults. Additional data concerning absolute skeletal muscle was also provided.

### Physical function

Walking speed was evaluated over a 10-meter distance that was clearly marked out on the ground. An additional initial distance of 2 m was also marked out, thus making an overall marked-out distance of 12 m in length. The participants were asked to walk the entire distance at their usual pace. The time required to complete the inner 10-m distance was assessed.^10^ Walking speed was calculated as a continuous value.

Cutoffs of 0.8 m/s and 1.0 m/s cutoffs were applied. The value of 0.8 m/s has been suggested in other studies as representative of slow walking. However, considering the range in walking speed that is seen in such studies, and the characteristics of the sample that we studied, 1 m/s was also used.^2^^,^^3^

The timed up-and-go (TUG) test has been widely described. The time taken for the subject to stand up from a seated position on a chair, walk three meters ahead in a straight line, go round a cone and return to the chair and sit down is measured.^11^

Handgrip strength was measured using a Jamar digital dynamometer (Jamar Plus+; Sammons Preston, Rolyon, Bolingbrook, IL, USA). While seated, the subject held the dynamometer with elbow flexed at 90° without it touching his/her body. After preparation, the subject was instructed to pull the lever as much as possible. Each hand was tested once and the best value was used in the analysis. The subjects were also instructed to avoid the Valsalva maneuver or blocked breath while performing the test. Handle position two was set as standard for all subjects, as previously recommended.^12^

### Statistical analyses

All analyses were carried out using the SPSS software, version 21.0 (IBM Inc., Chicago, IL, USA) and the MedCalc statistical software version 17.2 (MedCalc Software, Ostend, Belgium). Values were presented descriptively, as the mean ± standard deviation (SD) for continuous variables and frequency (%) for categorical values.

To compare the older adults’ characteristics according to gender, unpaired t tests and chi-square tests were used for continuous and categorical variables, respectively. In analyses according to age, the subjects were divided into five groups (60 to 64, 65 to 69, 70 to 74, 75 to 79 and 80 plus years old). For continuous variables, analysis of variance (ANOVA) was used; when statistical differences were found, Tukey’s post-hoc test was applied. For categorical variables, the chi-square test or Fisher’s exact test was used.

In addition, receiver operating characteristic (ROC) curve analyses were used to ascertain cutoff values for ASM and handgrip strength in relation to fear of falling. For this, different adjustments of ASM and handgrip strength were used; the curves were then compared to investigate statistical differences among them. The ROC curve compares the true-positive rate (sensitivity) versus the false-positive rate (1 - specificity) across a range of values, regarding the ability to predict a dichotomous outcome. High sensitivity corresponds to high negative predictive value, while high specificity corresponds to high positive predictive value. Sensitivity and specificity were used to identify the cutoff values for ASM and handgrip strength in this study.^13^

The area under the curve (AUC) measures test performance and describes the probability that a test will correctly identify individuals who did or did not have a condition and who were randomly selected from the cohort. Generally, the closer the AUC is to 1, the better the overall diagnostic performance of the test will be, and the closer to 0.5 that it is, the poorer the test will be.^14^^,^^15^

Sensitivity, specificity, positive predictive value (PPV), negative predictive value (NPV) and likelihood ratios (positive [LR+] and negative [LR-]) for ASM and handgrip strength in relation to fear of falling were computed. Predictive values describe the probability that a person has a condition once the results of his or her tests are known. LR+ and LR- indicate what the odds are that a disease will increase or decrease when a test is positive or negative, respectively.

Fear of falling was selected as the primary outcome for this study because of its association with psychological and physical issues such as falls, loss of confidence, restriction of activities and social withdrawal, which may lead to dependence and disability.^16^^,^^17^ Other variables were considered as outcomes, such as falls and walking speed; however, due to the small number of subjects with positive results or missing data, no further analyses were conducted. In all analyses, statistical significance was set at P < 0.05.

## RESULTS

In total, 578 older adults (122 males and 456 females) participated in this study. These individuals presented diversity of race, ethnicity and geographic area of origin, and a range of health and functional states. Their characteristics are shown descriptively in Table 1. The mean age was 70.0 ± 6.7 years for males and 69.4 ± 6.6 for females. Women had lower strength and were more overweight than men. Moreover, more women experienced a fall event during the year prior to this investigation (women 25.3% and men 14.4%) and reported fear of falling (women 65.7% and men 43.7%). Regarding physical function, women had slow walking speed than men (Table 1).

**Table 1.**
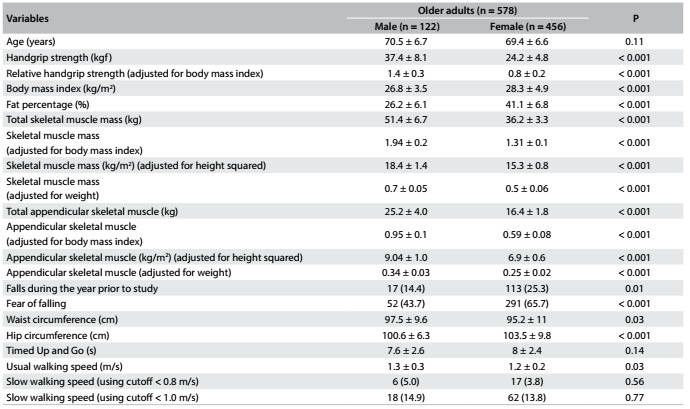
Participants’ characteristics

Values are mean ± standard deviation or n (%).

Tables 2 and 3 present the study data according to gender, divided into age groups. The highest rates of fear of falling were shown by the group aged 80 years and over, for both men and women. However, the difference was statistically significant only for men. Among the men, those aged 60 to 64 years were stronger than those aged 80 and over, regarding the absolute values of handgrip strength. This difference was not seen when the data were adjusted according to BMI. The age group from 60 to 64 years also had higher levels of skeletal muscle (total and adjusted according to height squared) and total ASM. Regarding walking speed and the TUG tests, function also decreased as age increased; a similar trend was observed regarding BMI, but not regarding fat percentage (Table 2).

**Table 2.**
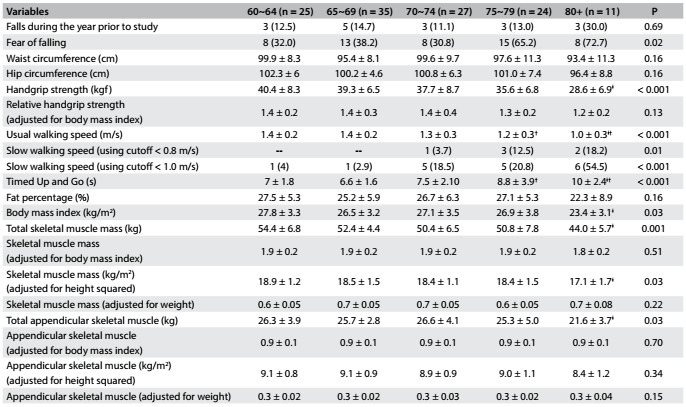
Characteristics of older men (n = 122) according to age

Values are mean ± standard deviation or n (%). Tukey’s post-hoc test: ǂ: ≠ 60~64; †: ≠ 65~69.

**Table 3.**
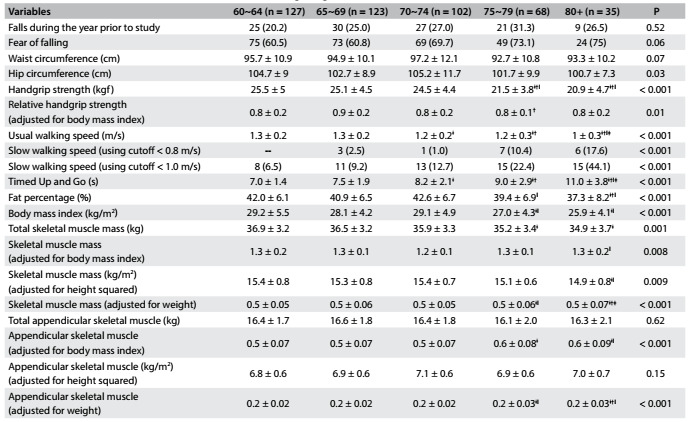
Characteristics of older women (n = 455) according to age

Values are mean ± standard deviation or n (%). Tukey’s post-hoc test: ǂ: ≠ 60~64; †: ≠ 65~69; ‖: ≠ 70~74; ‡: ≠ 75~79.

It was evident that older women had slower walking speed and TUG, and lower muscle strength than younger women, as shown by both absolute and relative handgrip strength. Fat percentage, BMI and skeletal muscle (total and adjusted according to BMI, height squared and weight) also decreased with increasing age. Regarding ASM, only the total value and the value adjusted according to height squared failed to show statistical differences (Table 3).

**Figure 1.**
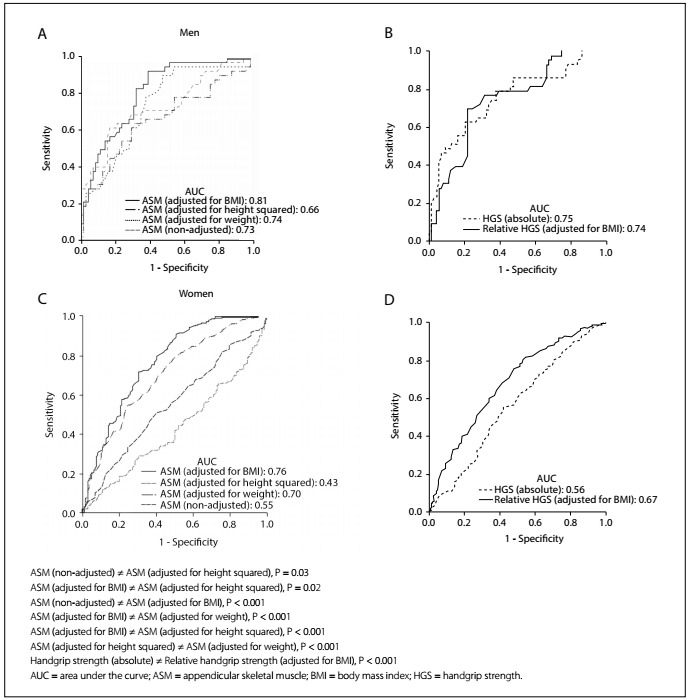
Receiver operating characteristic curves for appendicular skeletal muscle with different adjustments and for handgrip strength (absolute value and adjusted for body mass index). Fear of falling was used as the outcome variable. Data on men (A-B) and women (C-D) are presented. Statistical differences among or between curves are presented, as applicable.

The ROC curves and comparisons among them are presented in Figure 1. Regarding ASM, the adjustment according to BMI showed the best AUC in relation to fear of falling. Cutoff values were identified both for men and for women. For men, as shown in Table 4, the cutoff was 0.85 (AUC = 0.81; 95% confidence interval, CI = 0.73-0.89; P < 0.001). Table 5 shows the accuracy data for ASM for men and Table 6 for women. For women (Table 7), the cutoff was 0.53 (AUC = 0.76; 95% CI = 0.71-0.81; P < 0.001).

**Table 4.**
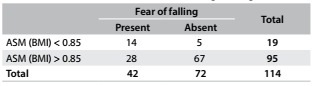
Appendicular skeletal muscle (ASM) adjusted for body mass index (BMI), in relation to fear of falling among men

Pearson chi-square P < 0.001.

**Table 5.**
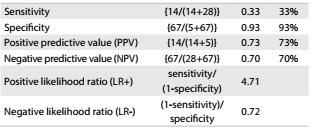
Accuracy of appendicular skeletal muscle (ASM) for prediction of fear of falling among men

**Table 6.**
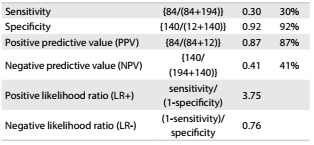
Accuracy of relative appendicular skeletal muscle (ASM) for prediction of fear of falling among women

**Table 7.**
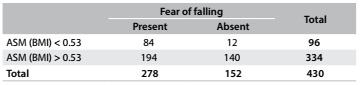
Appendicular skeletal muscle adjusted (ASM) for body mass index (BMI), in relation to fear of falling among women

Pearson chi-square P < 0.001.

Concerning handgrip strength, absolute values showed slight better AUC than did relative values among the men, while relative handgrip strength showed better AUC among the women. Therefore, we present cutoff values for both absolute and relative values: for men, as shown in Table 8, the cutoff for absolute handgrip strength was 30.0 kgf (AUC = 0.75; 95% CI = 0.66-0.84; P < 0.001). Table 9 shows the accuracy values for HGS for men, such as sensitivity, and Table 10 shows these values for women. The cutoff for women was 21.7 kgf (AUC = 0.56; 95% CI = 0.51-0.62; P = 0.02), as shown in Table 11. The cutoffs for relative handgrip strength were 1.07 (AUC = 0.74; 95% CI = 0.65-0.83; P < 0.001).

**Table 8.**
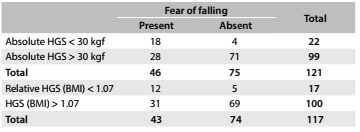
Absolute* and relative^†^ handgrip strength (HGS) in relation to fear of falling among men

*Pearson chi-square P < 0.001; ^†^Pearson chi-square P = 0.002.

**Table 9.**
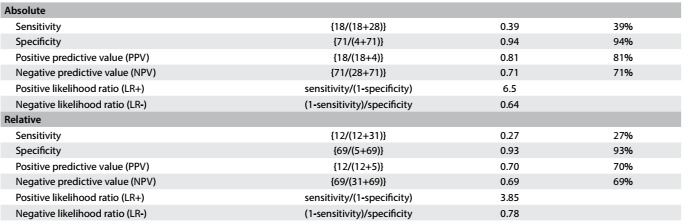
Accuracy of absolute and relative handgrip strength (HGS) for prediction of fear of falling among men

**Table 10.**
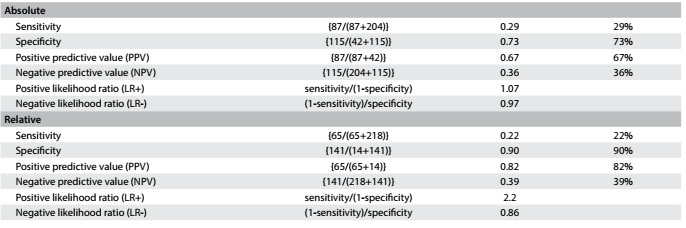
Accuracy of absolute and relative handgrip strength (HGS) for prediction of fear of falling among women

**Table 11.**
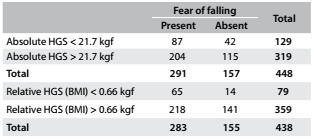
Absolute* and relative^†^ handgrip strength (HGS) in relation to fear of falling among women

*Pearson chi-square P = 0.48; ^†^Pearson chi-square P < 0.001.

## DISCUSSION

This study presented reference values for strength, physical function tests, body composition, anthropometric measurements, falls and fear of falling according to age from a community-based cohort of older men and women aged 60 years and over. Moreover, cutoff values for ASM and handgrip strength, which are useful for ascertaining the existence of sarcopenia in older adults, were also presented in relation to fear of falling.

As extensively reported in the literature, differences according to gender concerning ASM, strength and body composition were observed, as well as decline in physical function with increasing age among older adults. The tests used in this study have clinical relevance, and reference values of this nature are scarce in the Brazilian literature, which increases the external validity of this study. Even though some of these values were similar to those found in other populations, local values are preferable when available, given that regional characteristics can alter results and comparisons. These data may be useful for clinicians who need reference values to make comparisons with observed performance within clinical practice and research, and to compare different populations.

The evidence from this study highlights the imminent hazard that surrounds the oldest age group (80 years and over). These individuals showed the highest fear of falling, which may have impacted on their physical performance, which was the worst among the groups. It is difficult to predict when this cascade effect will begin, but it is crucial to implement interventions addressing physical and psychosocial issues, in order to face up to these conditions and thus promote health.

The values for handgrip strength that we found here were similar to those shown by Yoshimura et al.^18^ However, the subjects in their study performed better regarding walking speed. Importantly, in their study, the subjects were categorized into decades of age and walking speed was measured along a 6-m path.

The Asian Working Group for Sarcopenia (AWGS) recommended using the lowest 20^th^ percentile of handgrip strength of the study population as the cutoff value for low strength, due to the lack of outcome-based cutoff values. Thus, they suggested values of < 26 kgf for men and < 18 kgf for women.^2^ Similarly, the European Working Group on Sarcopenia in Older People (EWGSOP) suggested < 30 kgf for men and < 20 kgf for women as cutoff values.^3^ In our study, we found cutoffs of 30 kgf for Brazilian men and 21.7 kgf for Brazilian women, for absolute handgrip strength values. Although we were unable to contribute cutoff values for walking speed among Brazilian older adults at this time, both of these previous studies (AWGS and EWGSOP) recommended use of < 0.8 m/s as the cutoff for slow walking performance.^2^^,^^3^

Concerns have been raised regarding the influence of body mass on the relationships between performance, strength and muscle mass. These were raised especially by the Foundation for the National Institutes of Health (FNIH Sarcopenia Project), a large sample study that used multiple existing data sources to identify criteria for clinically relevant weakness and low lean mass.^5^^,^^19^^,^^20^ Therefore, we performed several analyses to clarify the need to adjust handgrip strength and muscle mass for body mass. Through this, we found cutoffs for relative handgrip strength adjusted for BMI of 1.07 for men and 0.66 for women. The definitions for weakness suggested from the FNIH Sarcopenia Project were ratios < 1.0 for men and < 0.56 for women.^20^ The necessity for this adjustment will be further discussed below.

Regarding the TUG, Bohannon (2006) conducted a descriptive meta-analysis and found mean values according to age (60 to 69, 70 to 79 and 80 to 99 years) of 8.1 seconds (95% CI = 7.1-9.0), 9.2 seconds (95% CI = 8.2-10.2) and 11.3 seconds (95% CI = 10.0-12.7), respectively.^11^ Individuals whose performance was outside the limits of these confidence intervals could be considered to have worse-than-average performance. These values were within the range that we found in our study. Furthermore, considering healthy Japanese individuals aged 60 years and over, Kamide et al. found that the weighted mean for TUG with maximum effort was 6.60 seconds (95% CI = 6.18-7.02) and that at the usual pace it was 8.86 seconds (95% CI = 7.99-9.72).^21^ This was certainly faster than has been found in other populations.

The data of our study showed through specific tests that physical performance declined for both genders as age increased, but that the changes in skeletal muscle depended on the adjustment applied. The ASM cutoff values (adjusted for BMI) that were identified among older adults according to gender were 0.85 for men and 0.53 for women. Interestingly, the values proposed by the FNIH Sarcopenia Project were 0.789 and 0.512, for men and women, respectively.^5^ We also found that adjustments for BMI were the best approach in relation to both genders. Therefore, we suggest that these cutoffs should be used to screen older adults of both sexes for higher risk of disability, in relation to fear of falling. These values provide a more realistic approach towards Brazilian older individuals.

Because of the adjustments to the data that we implemented, our results are not directly comparable with other proposed definitions for low ASM or sarcopenia. Initially, both the EWGSOP and AWGS groups suggested the approach of using -2 SD of ASM in young individuals as a cutoff point for muscle mass.^2^^,^^3^ However, low muscle mass alone is not consistently associated with adverse health outcomes,^5^ which thus poses the challenge of implementing new approaches. Hence, the methodology adopted in our study limited our ability to make comparisons, but it stimulates other researchers to provide more suitable and comparable data.

Considering the role of body mass, it differed according to gender. In men, the AUC was slightly smaller for relative than for absolute handgrip strength. However, in women, relative handgrip strength showed better results. Interestingly, Alley et al. reported a similar finding.^19^ In our study, this was seen despite our small sample size in the men’s group and with a different outcome-based variable. It remains unclear why this occurred. BMI would be more important for women than for men.

To our knowledge, this was the first study to provide reference data and cutoff values adjusted according to body mass, for Brazilian older adults. We expect that these data will be useful both for clinicians within their practice and for researchers, who will now be able to use Brazilian data regarding physical function and muscle mass in older adults.

We provided several adjustments to the data, but for consistency, we encourage researchers to use ASM adjusted for BMI and, according to convenience, to choose between absolute and relative handgrip strength adjusted for BMI, for both men and women, or even different types of indicators for each gender. For walking speed, a cutoff value < 0.8 m/s, as previously suggested,^2^^,^^3^^,^^5^ can be applied within both research and clinical practice to identify mobility impairment. Values for physical function tests and other variables can be used as references, according to age categories, as we presented in this study.

The limitations of this study included: (i) its cross-sectional design, which did not allow determination of a cause-effect relationship between the variables; (ii) the small number of older male subjects; (iii) the retrospective nature of the data on occurrences of falls, which may have been biased; and (iv) the use of fear of falling, and no other disability condition or mortality, as the outcome. Longitudinal analyses are preferable over cross-sectional designs and are appropriate for establishing clinical diagnostic cutoff values.^5^ Moreover, even though mortality or other disability outcomes seem more representative for sarcopenia, fear of falling was highly associated with sarcopenia among older adults,^22^ as previously verified, thus justifying its use as an outcome. We suggest that future studies should recruit larger numbers of male subjects and use different sampling fields and alternative methods for investigating body composition, such as dual-energy x-ray absorptiometry. In addition, longitudinal studies using disability or mortality as an outcome are necessary in order to determine optimal cutoffs for ASM, handgrip strength and walking speed.

In summary, we identified age-related decline in physical function and changes in body composition and anthropometric measurements. Moreover, cutoff values for handgrip strength (absolute: men < 30 kgf; women < 21.7 kgf; and relative: men < 1.07; women < 0.66) and for ASM (ASM adjusted for BMI: men < 0.85; women < 0.53), in relation to fear of falling among Brazilian older adults were also provided. Further analyses also suggested that adjustment for BMI may influence how the data can be interpreted. The cutoff value for walking speed was established as < 0.8 m/s, as previously recommended. In future studies, we intend to evaluate the capacity of these cutoff values to identify individuals who are in a vulnerable condition, especially regarding low quality of life and frailty.

## CONCLUSION

The values for physical function tests and other variables, presented according to age groups, highlight the hazards that surround the most elderly individuals. These data are useful references both for clinicians within practice and for researchers. Moreover, ASM adjusted for BMI was the best approach, while adjustment of handgrip strength varied according to gender. We recommend use of ASM adjusted for BMI and to choose either absolute handgrip strength or relative handgrip strength (adjusted for BMI), for both men and women, according to study needs.
